# Synthesis, crystal structure and Hirshfeld surface analysis of 2-(ethyl­sulfan­yl)-3-(2-methyl­prop­yl)-5,5-diphenyl-4,5-di­hydro-3*H*-imidazol-4-one

**DOI:** 10.1107/S2056989026008790

**Published:** 2026-09-03

**Authors:** Abderrazzak El Moutaouakil Ala Allah, Chiara Massera, Abdulsalam Alsubari, Joel T. Mague, Youssef Ramli

**Affiliations:** a Laboratory of Medicinal Chemistry Drug Sciences Research Center Faculty of Medicine and Pharmacy Mohammed V University in Rabat Morocco; b Dipartimento di Scienze Chimiche, della Vita e della Sostenibilità Ambientale, Università di Parma, Parco Area delle Scienze 17/A, 43124 Parma, Italy; c Laboratory of Medicinal Chemistry, Faculty of Clinical Pharmacy, 21 September University for Medical and Applied Sciences, Yemen; d Department of Chemistry, Tulane University, New Orleans, LA 70118, USA; Katholieke Universiteit Leuven, Belgium

**Keywords:** crystal structure, imidazolone, supra­molecular inter­actions, Hirshfeld surface analysis

## Abstract

In the title compound, C_21_H_24_N_2_OS, are reported. The imidazolone ring is essentially planar, and the coordination geometry about the N-substituted nitro­gen atom is also nearly planar, consistent with partial π-delocalization involving its lone pair. The two phenyl rings are markedly inclined to the imidazolone plane, with dihedral angles of 74.98 (9) and 59.67 (8)°. In the crystal, weak C—H⋯O and C—H⋯π inter­actions contribute to the three-dimensional packing arrangement. Hirshfeld surface analysis shows that H⋯H contacts dominate the inter­molecular inter­actions, accounting for 68.5% of the surface, followed by C⋯H/H⋯C (19.1%) and O⋯H/H⋯O (6.1%) contacts. A comparison with related structures in the Cambridge Structural Database indicates that the geometric parameters of the heterocyclic core are consistent with those of closely related derivatives.

## Abstract 2

In the title mol­ecule, the imidazolone ring is essentially planar and the inclinations of the planes of the two attached phenyl groups to its plane are distinctly different. There is a degree of π-delocalization in the ring about the tertiary nitro­gen which involves its lone pair. Directed inter­molecular inter­actions comprise C—H⋯O hydrogen bonds and two sets of C—H⋯π(ring) inter­actions. A Hirshfeld surface analysis was performed.

## 1. Chemical context

Hydantoin, C_3_H_4_N_2_O_2_, is a non-aromatic five-membered heterocycle that is regarded as a privileged scaffold of considerable importance in medicinal chemistry (Palkhede *et al.*, 2026 ▸). The significance of the hydantoin core in drug discovery is highlighted by its presence in several clinically used drugs, including phenytoin, nitro­furan­toin, and enzalutamide (Shinde *et al.*, 2026 ▸; El Moutaouakil Ala Allah *et al.*, 2024*a* ▸). Furthermore, hydantoin derivatives have demonstrated therapeutic potential against a wide range of diseases, exhibiting anti­viral (Sefrji *et al.*, 2026 ▸), anti­diabetic (Guerrab *et al.*, 2025 ▸; El Moutaouakil Ala Allah *et al.*, 2025*a* ▸), anti­microbial (El Moutaouakil Ala Allah *et al.*, 2024*a* ▸, 2026**a* ▸)*, and anti­epileptic activities (El Moutaouakil Ala Allah *et al.*, 2024*b* ▸). More recently, the hydantoin scaffold has also emerged as an efficient corrosion inhibitor for steel substrates (Belkheiri *et al.*, 2024 ▸; Dahmani *et al.*, 2025 ▸; Safir *et al.*, 2025 ▸). This work is part of our ongoing research program devoted to the chemistry of hydantoin derivatives (El Moutaouakil Ala Allah *et al.*, 2023 ▸; Guerrab *et al.*, 2022 ▸, 2023 ▸). Herein, we report the chemical synthesis and crystal structure of the title compound, C_21_H_24_N_2_OS, (**3**).
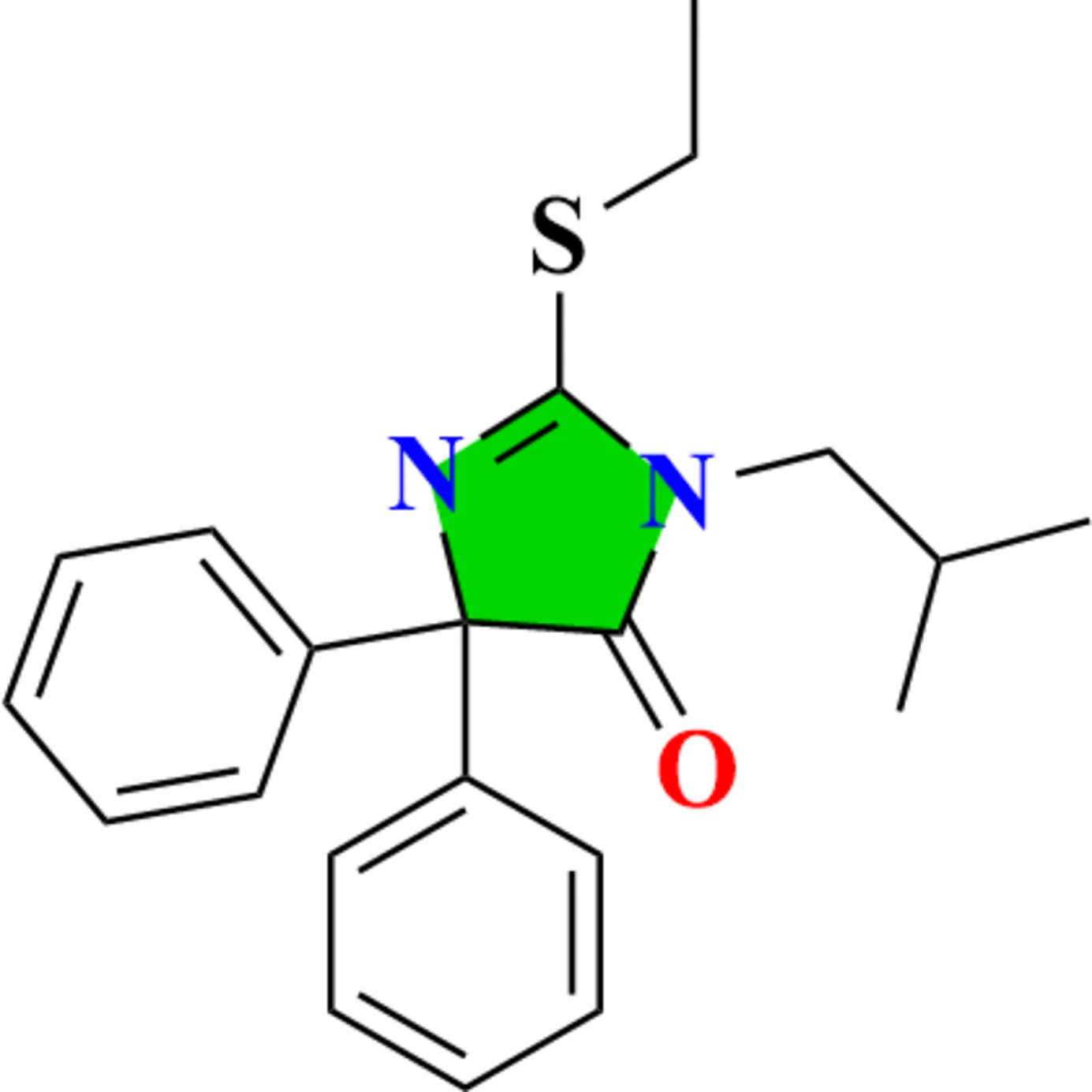


## 2. Structural commentary

**Figure 1:**
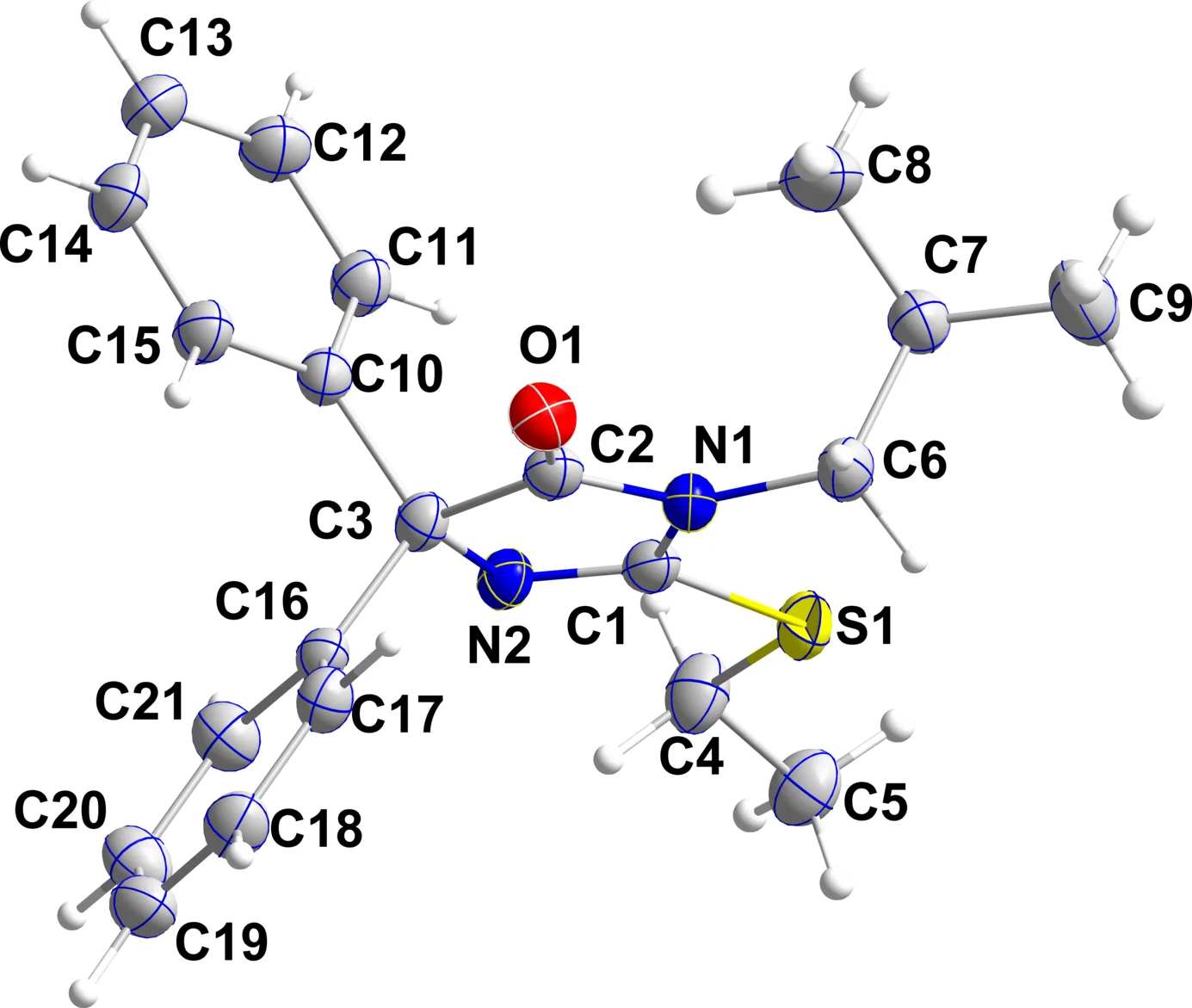
Perspective view of the title mol­ecule with labeling scheme and 40% probability displacement ellipsoids.

In the title mol­ecule (Fig. 1 ▸), the imidazolone ring is essentially planar, with a maximum deviation from the mean plane of 0.010 (2) Å for C1. Atom C6 lies 0.049 (2) Å from this plane. The coordination about N1 is essentially planar, indicating involvement of its lone pair in N→C π-bonding. This is reflected in the N1—C1 and N1—C2 bond lengths of 1.397 (2) and 1.3749 (19) Å, respectively, which are shorter than a typical C*sp*^2^—N*sp*^3^bond but longer than the formal C1=N2 of 1.281 (2) Å. The dihedral angles between the mean plane of the imidazolone ring and those of the C10–C15 and the C16–C21 benzene rings are 74.98 (9) and 59.67 (8)°, respectively. The C4—S1—C1—N1 torsion angle of −178.78 (12)° indicates the α carbon atom of the ethyl group lies essentially in the plane of the imidazolone ring, which is a common feature of the alkyl- or aryl­thio group in this class of compounds. By contrast, the C1—N1—C6—C7 torsion angle is 76.9 (2)°, placing the isobutyl group approximately perpendicular to the imidazolone plane.

## 3. Supra­molecular features

**Figure 2:**
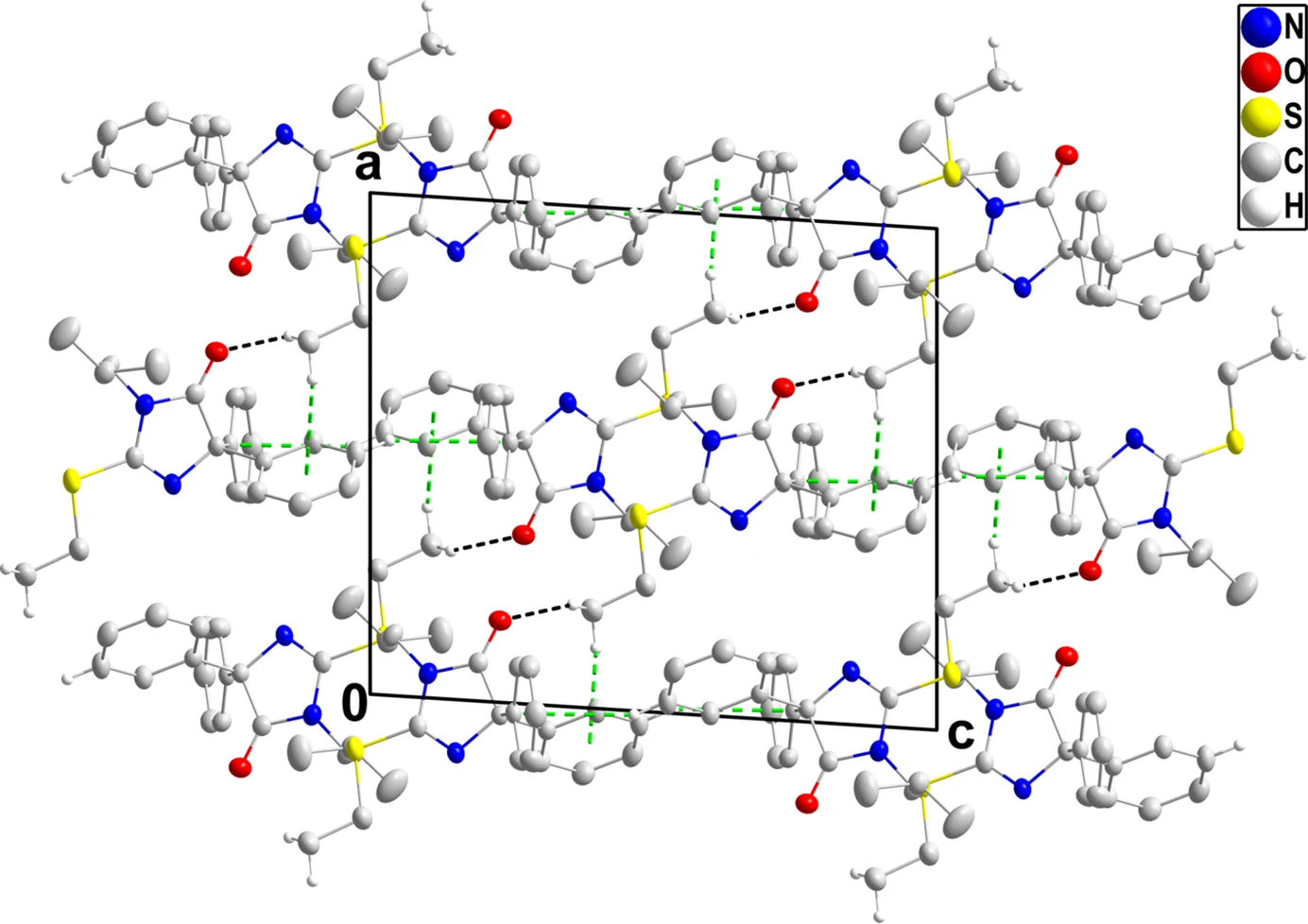
Packing viewed along the *b*-axis direction with C—H⋯O and C—H⋯π(ring) inter­actions depicted by black and green dashed lines, respectively. Hydrogen atoms not involved in these inter­actions are omitted for clarity.

The crystal packing features C5—H5*C*⋯O1^ii^ hydrogen bonds together with C5—H5*A*⋯*Cg*2^i^ inter­actions (Table 1 ▸), which generate chains of mol­ecules extending parallel to [10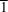]. These chains are connected by C14—H14⋯*Cg*3^iii^ inter­actions (Table 1 ▸), forming the three-dimensional crystal packing (Fig. 2 ▸).

## 4. Database survey

**Figure 3:**
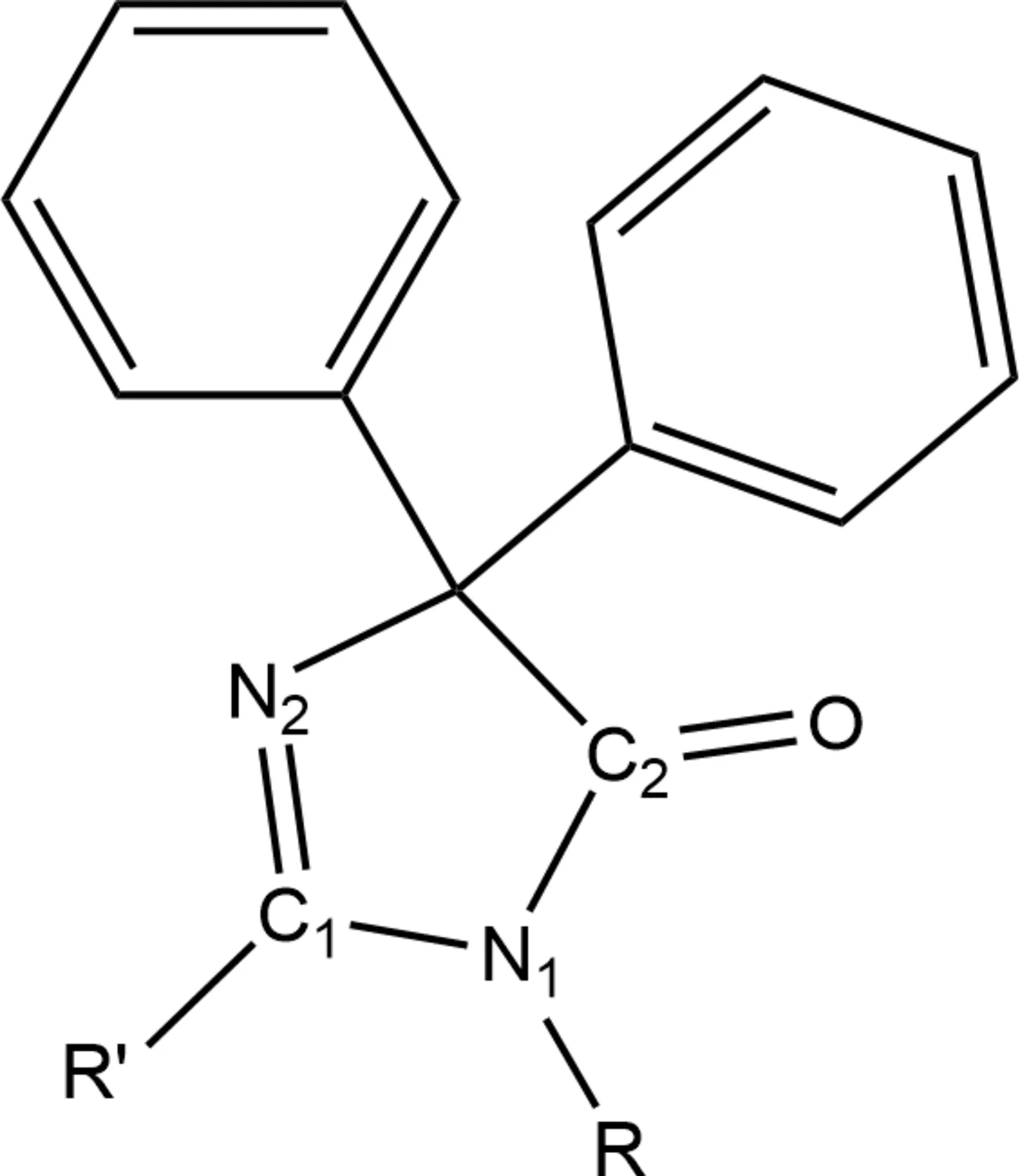
The search fragment used in the database survey (*R* = C, *R*′ = any atom or group).

A search of the Cambridge Structural Database (CSD, updated to June 2026; Groom *et al.*, 2016 ▸) with the fragment pictured in Fig. 3 ▸ (*R* = C, *R*′ = any atom or group) returned 22 hits of which 14 were considered similar to the title mol­ecule. Of the remainder, two had connections between the two phenyl rings and the rest had the substituents on N1 and C1 as part of a ring (*e.g. RR*′ = –CH_2_CH_2_S–, CSD refcode DIYRAE: Karolak-Wojciechowska *et al.*, 1985 ▸), which would markedly affect their conformations and so were considered not to be strictly comparable to the title mol­ecule. Table 2 ▸ lists the 14 hits and the title mol­ecule together with selected structural data for comparison. Since the two phenyl groups are attached to the saturated carbon atom of the imidazolone ring, there is no electronic factor to influence the rotational orientations of these rings. Thus, the inclinations of their mean planes to that of the imidazolone ring will reflect a balance between the intra­molecular and inter­molecular contacts made by the two rings. This can result in the dihedral angles being markedly different, as in EHEGIL, or very nearly the same, as in YEYYUA (Table 2 ▸). In the title mol­ecule, the former is the case. The C2—N1 and C1—N1 bond lengths in the title compound are generally comparable to those observed in the related structures, consistent with a similar degree of π-delocalization. REFVIH is the only notable exception, possibly owing to the influence of the basic di­methyl­amino substituent.

## 5. Hirshfeld surface analysis

**Figure 4:**
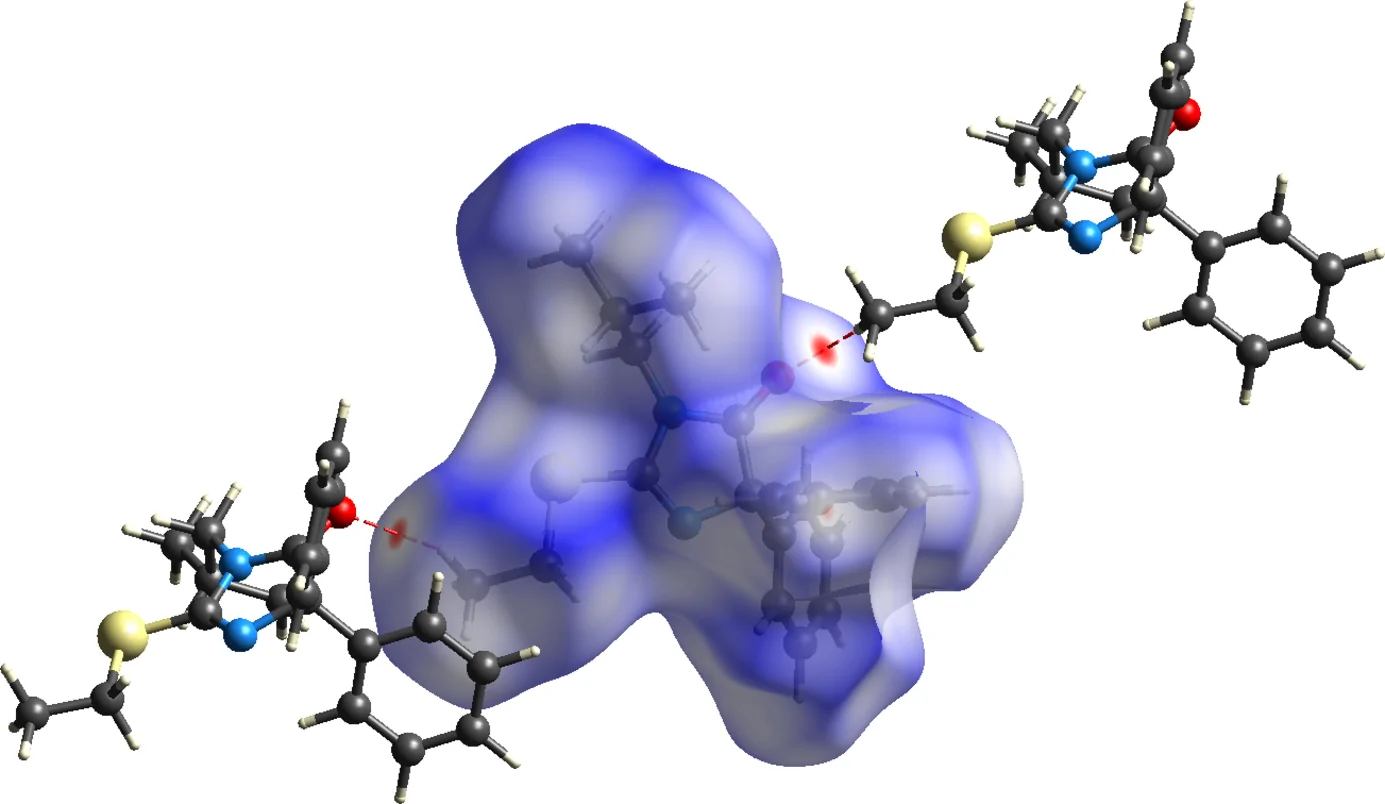
The *d*_norm_ Hirshfeld surface for the title mol­ecule with two neighboring mol­ecules included. The C—H⋯O hydrogen bonds are depicted by dashed lines.

**Figure 5:**
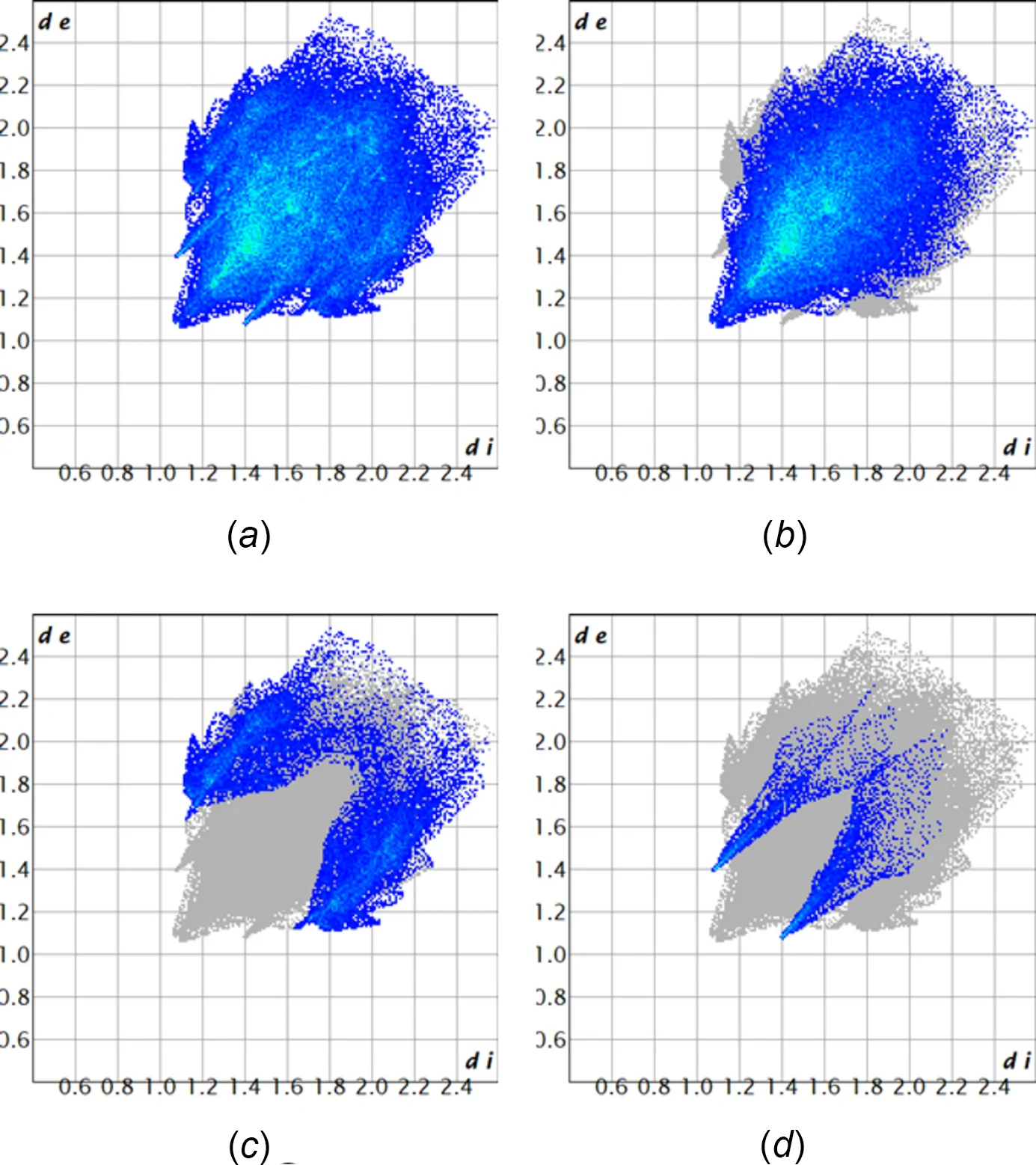
Two-dimensional fingerprint plots for the title mol­ecule showing all inter­molecular inter­actions (*a*) and those delineated into H⋯H (*b*), C⋯H/H⋯C (*c*) and O⋯H/H⋯O contacts (*d*).

The Hirshfeld *d*_norm_ surface of the title mol­ecule was calculated using *CrystalExplorer* (Spackman *et al.*, 2021 ▸). The inter­pretation of Hirshfeld surfaces and associated fingerprint plots has been described in detail by Tan *et al.* (2019 ▸). Fig. 4 ▸ shows the *d*_norm_ surface together with two neighboring mol­ecules involved in close C—H⋯O hydrogen bonds (Table 1 ▸ and Fig. 2 ▸). Fig. 5 ▸ shows the two-dimensional fingerprint plots where the H⋯H contacts (Fig. 5 ▸*b*) account for 68.5% of the inter­molecular contacts, as expected from the hydrogen-rich mol­ecular periphery. The C⋯H/H⋯C inter­actions (Fig. 5 ▸*c*) contribute 19.1% of the total and are due largely to the two sets of C—H⋯π(ring) inter­actions (Table 1 ▸). The O⋯H/H⋯O contacts (Fig. 5 ▸*d*) are largely due to the C—H⋯O hydrogen bonds and appear as a pair of sharp peaks with a contribution of 6.1%. All remaining contact types together contribute approximately 6.3%.

## 6. Synthesis and crystallization

**Figure 6:**
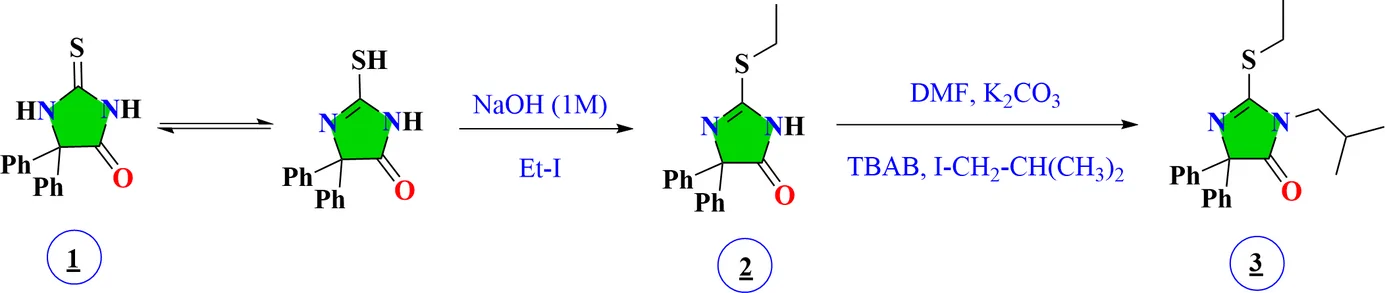
Reaction sequence for the synthesis of title compound (**3**).

The reaction sequence is shown in Fig. 6 ▸. Thio­hydantoin **1** was obtained by the condensation of benzil with thio­urea according to the reported procedure (Dahmani *et al.*, 2024 ▸). Subsequently, a selective *S*-alkyl­ation reaction was carried out using a 1 *M* aqueous sodium hydroxide solution and ethyl iodide, affording compound **2** exclusively. (The reaction was monitored by TLC and showed a single spot corresponding to compound **2**.) Thereafter, isobutyl iodide was introduced to perform an *N*-alkyl­ation reaction, thereby substituting the nitro­gen atom at the N3 position with an isobutyl group, following a procedure similar to that previously reported by our research group (El Moutaouakil Ala Allah *et al.*, 2026*a* ▸; El Moutaouakil Ala Allah *et al.*, 2024*b* ▸). The solid obtained upon the synthesis was recrystallized from ethanol solution to afford thick, colorless, plate-like crystals of the title compound **3**.

Yield: 90%; m.p. = 411–413 K; FT-IR (ATR, cm^−1^): 3062 (C—H Ar), 2856 (C—H aliphatic), 1724 (C=O); ^1^H NMR (500 MHz, CDCl_3_): δ ppm 0.86 [*d*, 6H, N—CH_2_—CH(CH_3_)_2_], 1.38 (*t*, 3H, S—CH_2_—CH_3_), 1.52 [*m*, 1H, N—CH_2_—CH(CH_3_)_2_], 3.18 (*q*, 2H, S—CH_2_—CH_3_), 4.52 [*d*, 2H, N—CH_2_—CH(CH_3_)_2_], 7.22-7.50 (*m*, 10H, Ar H); ^13^C NMR (125 MHz, CDCl_3_): 13.95 (S—CH_2_—CH_3_), 20.96 [N—CH_2_—CH(CH_3_)_2_], 24.02 [N—CH_2_—CH(CH_3_)_2_], 24.48 (S—CH_2_—CH_3_), 48.56 [N—CH_2_—CH(CH_3_)_2_], 76.42 (C—2Ph), 126.98, 127.34, 128.15, 140.46 (C—Ar), 160.32 (C=N), 178.58 (C=O).

## 7. Refinement

Crystal data, data collection and structure refinement details are summarized in Table 3 ▸. The carbon-bound H atoms were placed in calculated positions and refined using a riding model, with C—H distances ranging from 0.95 to 1.00 Å and *U*_iso_(H) = 1.2*U*_eq_(C), or 1.5*U*_eq_(C) for methyl groups.

## Supplementary Material

Crystal structure: contains datablock(s) I. DOI: 10.1107/S2056989026008790/vm2335sup1.cif

Structure factors: contains datablock(s) I. DOI: 10.1107/S2056989026008790/vm2335Isup2.hkl

Supporting information file. DOI: 10.1107/S2056989026008790/vm2335Isup3.cml

CCDC reference: 2583088

Additional supporting information:  crystallographic information; 3D view; checkCIF report

## Figures and Tables

**Table 1:** Hydrogen-bond geometry (Å, °) *Cg*2 and *Cg*3 are the centroids of the C10–C15 and C16–C21 benzene rings, respectively.

*D*—H⋯*A*	*D*—H	H⋯*A*	*D*⋯*A*	*D*—H⋯*A*
C5—H5*A*⋯C*g*2^i^	0.98	2.90	3.670 (2)	136
C5—H5*C*⋯O1^ii^	0.98	2.57	3.487 (2)	156
C14—H14⋯C*g*3^iii^	0.95	2.79	3.6536 (17)	151

Symmetry codes: (i) 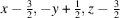; (ii) 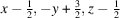; (iii) 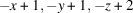.

**Table 2:** Results of database search (search fragment shown in Fig. 3 ▸)

Compound	*R*	*R*′	Dihedral angles (°)	C2—N1*^*a*^* (Å)	C1—N1*^*a*^* (Å)	Reference
**3**	*i*-Bu	SEt	74.98 (9), 59.67 (8)	1.375 (2)	1.397 (2)	This work
DACXAL	CH_2_C_2_H	SCH_2_C_2_H	68.89 (5), 54.67 (6)	1.3766 (13)	1.4026 (13)	El Moutaouakil Ala Allah *et al.* (2025*a* ▸)
EHEGIL	Et	SMe	83.53 (8), 59.50 (7)	1.3724 (18)	1.3959 (19)	El Moutaouakil Ala Allah *et al.* (2025*b* ▸)
HOPQAI	Et	SEt	89.59 (6), 54.66 (6)	1.3724 (14)	1.3959 (13)	El Moutaouakil Ala Allah *et al.* (2024*c* ▸)
RAHGUF	CH_2_Ph	SCH_2_Ph	72.05 (15), 66.59 (15), 69.00 (15), 58.50 (15)	1.375 (2), 1.380 (3)	1.399 (2), 1.402 (2)	Akrad *et al.* (2017 ▸)
REFVIH	*i*-Pr	NMe_2_	66.9 (6), 57.6 (6)	1.321 (12)	1.452 (14)	Mazik *et al.* (1996 ▸)
RIJZIW	*n*-Pr	(*n*-Pr)S	79.23 (6), 69.81 (6)	1.3685 (14)	1.4011 (14)	Akrad *et al.* (2018 ▸)
ROLJAH	all­yl	*S*(all­yl)	65.27 (6), 54.82 (6)	1.3738 (14)	1.3990 (14)	El Moutaouakil Ala Allah *et al.* (2024*c* ▸)
WOVJIE	CH_2_COOEt	SMe	69.52 (13), 67.03 (8)	1.371 (3)	1.406 (2)	El Moutaouakil Ala Allah *et al.* (2024*b* ▸)
YEYYUA	Me	SMe	72.32 (7), 67.03 (8)	1.3699 (16)	1.3989 (17)	El Moutaouakil Ala Allah *et al.* (2023 ▸)
ZOKBOU	Ph	*^*b*^*	73.62 (15), 56.82 (15)	1.372 (3)	1.408 (3)	Chen *et al.* (2024 ▸)
ZOKBUA	Ph	*^*c*^*	71.24 (12), 53.76 (15)	1.389 (3)	1.415 (3)	Chen *et al.* (2024 ▸)
EZOYIF	vin­yl	SMe	70.15 (9), 66.01 (8)	1.3868 (19)	1.4082 (19)	El Moutaouakil Ala Allah *et al.* (2026*b* ▸)
OYURAF	Ph	Ph	65.90 (9), 53.22 (9)	1.390 (3)	1.405 (3)	Goswami *et al.* (2026 ▸)
OYUREJ	Ph	4-ClC_6_H_4_	76.05 (6), 66.78 (6)	1.3898 (14)	1.4172 (15)	Goswami *et al.* (2026 ▸)

Notes: (*a*) Atom labels refer to Fig. 3 ▸; (*b*) 4-(10*H*-pheno­thia­zine)phenyl; (*c*) 10-phenyl-10*H*-pheno­thia­zin-3-yl.

**Table 3:** Experimental details

Crystal data	
Chemical formula	C_21_H_24_N_2_OS
*M*_r_	352.48
Crystal system, space group	Monoclinic, *P*2_1_/*n*
Temperature (K)	200
*a*, *b*, *c* (Å)	12.7849 (4), 10.1770 (4), 14.4793 (5)
β (°)	93.627 (1)
*V* (Å^3^)	1880.16 (11)
*Z*	4
Radiation type	Mo *K*α
μ (mm^−1^)	0.18
Crystal size (mm)	0.50 × 0.20 × 0.18

Data collection	
Diffractometer	Bruker D8 Venture PhotonIII
Absorption correction	Multi-scan (*SADABS*; Krause *et al.*, 2015 ▸)
*T*_min_, *T*_max_	0.368, 0.746
No. of measured, independent and observed [*I* > 2σ(*I*)] reflections	31797, 4662, 3601
*R*_int_	0.084
(sin θ/λ)_max_ (Å^−1^)	0.667

Refinement	
*R*[*F*^2^ > 2σ(*F*^2^)], *wR*(*F*^2^), *S*	0.049, 0.134, 1.05
No. of reflections	4662
No. of parameters	229
H-atom treatment	H-atom parameters constrained
Δρ_max_, Δρ_min_ (e Å^−3^)	0.37, −0.34

Computer programs: *APEX5* and *SAINT* (Bruker, 2016 ▸), *SHELXT2018/2* (Sheldrick, 2015*a* ▸), *SHELXL2019/3* (Sheldrick, 2015*b* ▸), *DIAMOND* (Brandenburg & Putz, 2012 ▸), *WinGX* (Farrugia, 2012 ▸), *publCIF* (Westrip, 2010 ▸) and *enCIFer* (Allen *et al.*, 2004 ▸).

## supplementary crystallographic information

### 2-(Ethylsulfanyl)-3-(2-methylpropyl)-5,5-diphenyl-4,5-dihydro-3H-imidazol-4-one Crystal data

**Table:**

C_21_H_24_N_2_OS	*F*(000) = 752
*M**_r_* = 352.48	*D*_x_ = 1.245 Mg m^−^^3^
Monoclinic, *P*2_1_/*n*	Mo *K*α radiation, λ = 0.71073 Å
*a* = 12.7849 (4) Å	Cell parameters from 876 reflections
*b* = 10.1770 (4) Å	θ = 2.1–28.3°
*c* = 14.4793 (5) Å	µ = 0.18 mm^−^^1^
β = 93.627 (1)°	*T* = 200 K
*V* = 1880.16 (11) Å^3^	Prismatic, colourless
*Z* = 4	0.5 × 0.2 × 0.18 mm

### 2-(Ethylsulfanyl)-3-(2-methylpropyl)-5,5-diphenyl-4,5-dihydro-3H-imidazol-4-one Data collection

**Table:**

Bruker D8 Venture PhotonIII diffractometer	4662 independent reflections
Radiation source: fine-focus sealed tube	3601 reflections with *I* > 2σ(*I*)
Graphite monochromator	*R*_int_ = 0.084
phi & ω scan	θ_max_ = 28.3°, θ_min_ = 2.1°
Absorption correction: multi-scan (SADABS; Krause *et al*., 2015)	*h* = −15→17
*T*_min_ = 0.368, *T*_max_ = 0.746	*k* = −13→13
31797 measured reflections	*l* = −17→19

### 2-(Ethylsulfanyl)-3-(2-methylpropyl)-5,5-diphenyl-4,5-dihydro-3H-imidazol-4-one Refinement

**Table:**

Refinement on *F*^2^	Primary atom site location: dual
Least-squares matrix: full	Secondary atom site location: difference Fourier map
*R*[*F*^2^ > 2σ(*F*^2^)] = 0.049	Hydrogen site location: inferred from neighbouring sites
*wR*(*F*^2^) = 0.134	H-atom parameters constrained
*S* = 1.05	*w* = 1/[σ^2^(*F*_o_^2^) + (0.0615*P*)^2^ + 0.5162*P*] where *P* = (*F*_o_^2^ + 2*F*_c_^2^)/3
4662 reflections	(Δ/σ)_max_ = 0.001
229 parameters	Δρ_max_ = 0.37 e Å^−^^3^
0 restraints	Δρ_min_ = −0.34 e Å^−^^3^

### 2-(Ethylsulfanyl)-3-(2-methylpropyl)-5,5-diphenyl-4,5-dihydro-3H-imidazol-4-one Special details

**Table:**

Geometry. All esds (except the esd in the dihedral angle between two l.s. planes) are estimated using the full covariance matrix. The cell esds are taken into account individually in the estimation of esds in distances, angles and torsion angles; correlations between esds in cell parameters are only used when they are defined by crystal symmetry. An approximate (isotropic) treatment of cell esds is used for estimating esds involving l.s. planes.

### 2-(Ethylsulfanyl)-3-(2-methylpropyl)-5,5-diphenyl-4,5-dihydro-3H-imidazol-4-one Fractional atomic coordinates and isotropic or equivalent isotropic displacement parameters (Å^2^)

**Table:**

	*x*	*y*	*z*	*U*_iso_*/*U*_eq_	
N1	0.54858 (11)	0.66344 (13)	0.60206 (9)	0.0278 (3)	
N2	0.39377 (10)	0.58903 (13)	0.65127 (8)	0.0274 (3)	
O1	0.66318 (9)	0.62966 (12)	0.72927 (8)	0.0342 (3)	
S1	0.38764 (3)	0.67250 (4)	0.47352 (3)	0.03519 (15)	
C1	0.44178 (13)	0.63817 (15)	0.58436 (10)	0.0271 (3)	
C2	0.57631 (12)	0.62265 (15)	0.69077 (10)	0.0269 (3)	
C3	0.47439 (12)	0.57100 (15)	0.72888 (10)	0.0258 (3)	
C4	0.25214 (14)	0.6282 (2)	0.48617 (12)	0.0411 (4)	
H4A	0.248702	0.542891	0.518714	0.049*	
H4B	0.218197	0.695522	0.523524	0.049*	
C5	0.19510 (14)	0.6182 (2)	0.39164 (12)	0.0412 (4)	
H5A	0.120318	0.603126	0.398735	0.062*	
H5B	0.223838	0.544775	0.357484	0.062*	
H5C	0.204193	0.700076	0.357489	0.062*	
C6	0.62030 (13)	0.72361 (16)	0.53972 (11)	0.0324 (4)	
H6A	0.607960	0.684392	0.477409	0.039*	
H6B	0.693230	0.702931	0.562056	0.039*	
C7	0.60814 (14)	0.87310 (17)	0.53198 (11)	0.0335 (4)	
H7A	0.534360	0.891899	0.508664	0.040*	
C8	0.6265 (2)	0.9401 (2)	0.62452 (14)	0.0579 (6)	
H8A	0.577319	0.905350	0.667684	0.087*	
H8B	0.615534	1.034913	0.617007	0.087*	
H8C	0.698502	0.923590	0.648995	0.087*	
C9	0.6801 (2)	0.9259 (2)	0.46124 (17)	0.0642 (7)	
H9A	0.666652	0.879137	0.402507	0.096*	
H9B	0.753260	0.912580	0.483756	0.096*	
H9C	0.666918	1.019901	0.451788	0.096*	
C10	0.45030 (12)	0.65603 (15)	0.81209 (10)	0.0257 (3)	
C11	0.38800 (13)	0.76706 (16)	0.80080 (11)	0.0315 (4)	
H11	0.357788	0.789197	0.741197	0.038*	
C12	0.36958 (15)	0.84610 (18)	0.87632 (12)	0.0377 (4)	
H12	0.326397	0.921698	0.868114	0.045*	
C13	0.41353 (15)	0.81557 (18)	0.96311 (12)	0.0379 (4)	
H13	0.400307	0.869573	1.014598	0.045*	
C14	0.47704 (15)	0.70581 (18)	0.97485 (11)	0.0379 (4)	
H14	0.508293	0.685140	1.034349	0.046*	
C15	0.49503 (14)	0.62613 (17)	0.89990 (10)	0.0323 (4)	
H15	0.538165	0.550538	0.908400	0.039*	
C16	0.48008 (12)	0.42469 (15)	0.75309 (9)	0.0252 (3)	
C17	0.57366 (13)	0.35568 (17)	0.76168 (10)	0.0302 (4)	
H17	0.637946	0.400188	0.754477	0.036*	
C18	0.57412 (14)	0.22169 (17)	0.78077 (11)	0.0350 (4)	
H18	0.638608	0.175096	0.785975	0.042*	
C19	0.48140 (15)	0.15620 (17)	0.79216 (12)	0.0379 (4)	
H19	0.481744	0.064505	0.804451	0.046*	
C20	0.38766 (14)	0.22508 (18)	0.78555 (13)	0.0386 (4)	
H20	0.323665	0.180729	0.794339	0.046*	
C21	0.38704 (13)	0.35831 (17)	0.76619 (12)	0.0339 (4)	
H21	0.322500	0.404829	0.761809	0.041*	

### 2-(Ethylsulfanyl)-3-(2-methylpropyl)-5,5-diphenyl-4,5-dihydro-3H-imidazol-4-one Atomic displacement parameters (Å^2^)

**Table:**

	*U*^11^	*U*^22^	*U*^33^	*U*^12^	*U*^13^	*U*^23^
N1	0.0326 (7)	0.0267 (7)	0.0245 (6)	−0.0009 (5)	0.0045 (5)	0.0011 (5)
N2	0.0309 (7)	0.0293 (7)	0.0217 (6)	0.0022 (5)	−0.0012 (5)	0.0019 (5)
O1	0.0283 (6)	0.0370 (7)	0.0371 (6)	−0.0009 (5)	0.0003 (5)	0.0014 (5)
S1	0.0438 (3)	0.0392 (3)	0.0220 (2)	−0.00504 (18)	−0.00225 (17)	0.00514 (16)
C1	0.0353 (9)	0.0224 (8)	0.0233 (7)	0.0021 (6)	0.0003 (6)	−0.0016 (6)
C2	0.0308 (8)	0.0235 (8)	0.0265 (7)	0.0025 (6)	0.0027 (6)	−0.0020 (6)
C3	0.0276 (8)	0.0285 (8)	0.0212 (7)	0.0017 (6)	0.0005 (6)	0.0012 (6)
C4	0.0395 (10)	0.0547 (12)	0.0287 (8)	−0.0013 (8)	−0.0010 (7)	0.0025 (8)
C5	0.0389 (10)	0.0498 (11)	0.0342 (9)	0.0050 (8)	−0.0029 (7)	−0.0003 (8)
C6	0.0378 (9)	0.0303 (9)	0.0302 (8)	0.0005 (7)	0.0112 (7)	0.0009 (6)
C7	0.0380 (9)	0.0304 (9)	0.0323 (8)	0.0002 (7)	0.0051 (7)	0.0024 (7)
C8	0.0931 (17)	0.0360 (11)	0.0438 (11)	−0.0006 (11)	−0.0015 (11)	−0.0059 (9)
C9	0.0847 (17)	0.0424 (12)	0.0696 (15)	−0.0062 (11)	0.0369 (13)	0.0097 (10)
C10	0.0269 (8)	0.0272 (8)	0.0232 (7)	−0.0014 (6)	0.0023 (6)	0.0007 (6)
C11	0.0342 (9)	0.0318 (9)	0.0284 (7)	0.0037 (7)	0.0019 (6)	0.0014 (6)
C12	0.0426 (10)	0.0323 (9)	0.0385 (9)	0.0065 (7)	0.0055 (8)	−0.0023 (7)
C13	0.0483 (11)	0.0348 (10)	0.0314 (8)	−0.0024 (8)	0.0087 (7)	−0.0057 (7)
C14	0.0505 (11)	0.0393 (10)	0.0237 (7)	−0.0003 (8)	−0.0006 (7)	0.0005 (7)
C15	0.0400 (9)	0.0315 (9)	0.0251 (7)	0.0030 (7)	0.0002 (6)	0.0016 (6)
C16	0.0306 (8)	0.0260 (8)	0.0187 (6)	0.0012 (6)	−0.0002 (6)	−0.0004 (5)
C17	0.0306 (8)	0.0342 (9)	0.0262 (7)	0.0026 (7)	0.0045 (6)	0.0005 (6)
C18	0.0397 (10)	0.0335 (9)	0.0319 (8)	0.0089 (7)	0.0028 (7)	0.0015 (7)
C19	0.0513 (11)	0.0274 (9)	0.0348 (9)	0.0002 (8)	−0.0002 (8)	0.0008 (7)
C20	0.0373 (10)	0.0351 (10)	0.0428 (9)	−0.0073 (7)	−0.0028 (7)	0.0050 (7)
C21	0.0299 (9)	0.0334 (9)	0.0379 (9)	0.0009 (7)	−0.0016 (7)	0.0034 (7)

### 2-(Ethylsulfanyl)-3-(2-methylpropyl)-5,5-diphenyl-4,5-dihydro-3H-imidazol-4-one Geometric parameters (Å, º)

**Table:**

N1—C2	1.3749 (19)	C9—H9A	0.9800
N1—C1	1.397 (2)	C9—H9B	0.9800
N1—C6	1.461 (2)	C9—H9C	0.9800
N2—C1	1.281 (2)	C10—C11	1.386 (2)
N2—C3	1.4873 (18)	C10—C15	1.394 (2)
O1—C2	1.2128 (19)	C11—C12	1.390 (2)
S1—C1	1.7426 (15)	C11—H11	0.9500
S1—C4	1.8107 (19)	C12—C13	1.379 (2)
C2—C3	1.539 (2)	C12—H12	0.9500
C3—C16	1.530 (2)	C13—C14	1.385 (3)
C3—C10	1.530 (2)	C13—H13	0.9500
C4—C5	1.513 (2)	C14—C15	1.386 (2)
C4—H4A	0.9900	C14—H14	0.9500
C4—H4B	0.9900	C15—H15	0.9500
C5—H5A	0.9800	C16—C17	1.386 (2)
C5—H5B	0.9800	C16—C21	1.391 (2)
C5—H5C	0.9800	C17—C18	1.391 (2)
C6—C7	1.533 (2)	C17—H17	0.9500
C6—H6A	0.9900	C18—C19	1.379 (3)
C6—H6B	0.9900	C18—H18	0.9500
C7—C8	1.509 (3)	C19—C20	1.386 (3)
C7—C9	1.518 (3)	C19—H19	0.9500
C7—H7A	1.0000	C20—C21	1.384 (2)
C8—H8A	0.9800	C20—H20	0.9500
C8—H8B	0.9800	C21—H21	0.9500
C8—H8C	0.9800		
			
C2—N1—C1	107.87 (13)	H8A—C8—H8C	109.5
C2—N1—C6	124.67 (13)	H8B—C8—H8C	109.5
C1—N1—C6	127.45 (13)	C7—C9—H9A	109.5
C1—N2—C3	106.10 (13)	C7—C9—H9B	109.5
C1—S1—C4	100.59 (8)	H9A—C9—H9B	109.5
N2—C1—N1	116.28 (13)	C7—C9—H9C	109.5
N2—C1—S1	126.40 (12)	H9A—C9—H9C	109.5
N1—C1—S1	117.30 (11)	H9B—C9—H9C	109.5
O1—C2—N1	125.88 (15)	C11—C10—C15	119.01 (14)
O1—C2—C3	128.80 (14)	C11—C10—C3	120.71 (13)
N1—C2—C3	105.30 (12)	C15—C10—C3	120.21 (14)
N2—C3—C16	108.29 (12)	C10—C11—C12	120.25 (15)
N2—C3—C10	111.14 (12)	C10—C11—H11	119.9
C16—C3—C10	112.26 (12)	C12—C11—H11	119.9
N2—C3—C2	104.42 (11)	C13—C12—C11	120.46 (16)
C16—C3—C2	112.66 (12)	C13—C12—H12	119.8
C10—C3—C2	107.83 (12)	C11—C12—H12	119.8
C5—C4—S1	109.56 (13)	C12—C13—C14	119.70 (16)
C5—C4—H4A	109.8	C12—C13—H13	120.1
S1—C4—H4A	109.8	C14—C13—H13	120.1
C5—C4—H4B	109.8	C13—C14—C15	120.06 (15)
S1—C4—H4B	109.8	C13—C14—H14	120.0
H4A—C4—H4B	108.2	C15—C14—H14	120.0
C4—C5—H5A	109.5	C14—C15—C10	120.51 (16)
C4—C5—H5B	109.5	C14—C15—H15	119.7
H5A—C5—H5B	109.5	C10—C15—H15	119.7
C4—C5—H5C	109.5	C17—C16—C21	118.83 (15)
H5A—C5—H5C	109.5	C17—C16—C3	122.81 (14)
H5B—C5—H5C	109.5	C21—C16—C3	118.36 (14)
N1—C6—C7	113.35 (13)	C16—C17—C18	120.48 (16)
N1—C6—H6A	108.9	C16—C17—H17	119.8
C7—C6—H6A	108.9	C18—C17—H17	119.8
N1—C6—H6B	108.9	C19—C18—C17	120.31 (16)
C7—C6—H6B	108.9	C19—C18—H18	119.8
H6A—C6—H6B	107.7	C17—C18—H18	119.8
C8—C7—C9	111.88 (18)	C18—C19—C20	119.56 (16)
C8—C7—C6	111.99 (15)	C18—C19—H19	120.2
C9—C7—C6	109.72 (15)	C20—C19—H19	120.2
C8—C7—H7A	107.7	C21—C20—C19	120.19 (16)
C9—C7—H7A	107.7	C21—C20—H20	119.9
C6—C7—H7A	107.7	C19—C20—H20	119.9
C7—C8—H8A	109.5	C20—C21—C16	120.61 (16)
C7—C8—H8B	109.5	C20—C21—H21	119.7
H8A—C8—H8B	109.5	C16—C21—H21	119.7
C7—C8—H8C	109.5		
			
C3—N2—C1—N1	−1.77 (18)	C16—C3—C10—C11	−144.20 (15)
C3—N2—C1—S1	176.61 (11)	C2—C3—C10—C11	91.13 (17)
C2—N1—C1—N2	2.02 (19)	N2—C3—C10—C15	160.33 (14)
C6—N1—C1—N2	−177.34 (14)	C16—C3—C10—C15	38.87 (19)
C2—N1—C1—S1	−176.51 (11)	C2—C3—C10—C15	−85.80 (17)
C6—N1—C1—S1	4.1 (2)	C15—C10—C11—C12	−0.8 (2)
C4—S1—C1—N2	2.86 (17)	C3—C10—C11—C12	−177.78 (15)
C4—S1—C1—N1	−178.78 (12)	C10—C11—C12—C13	0.4 (3)
C1—N1—C2—O1	−179.99 (15)	C11—C12—C13—C14	0.4 (3)
C6—N1—C2—O1	−0.6 (2)	C12—C13—C14—C15	−0.9 (3)
C1—N1—C2—C3	−1.22 (16)	C13—C14—C15—C10	0.5 (3)
C6—N1—C2—C3	178.16 (13)	C11—C10—C15—C14	0.3 (2)
C1—N2—C3—C16	−119.41 (13)	C3—C10—C15—C14	177.31 (15)
C1—N2—C3—C10	116.85 (14)	N2—C3—C16—C17	130.03 (14)
C1—N2—C3—C2	0.85 (16)	C10—C3—C16—C17	−106.90 (16)
O1—C2—C3—N2	179.00 (15)	C2—C3—C16—C17	15.06 (19)
N1—C2—C3—N2	0.28 (15)	N2—C3—C16—C21	−49.41 (17)
O1—C2—C3—C16	−63.7 (2)	C10—C3—C16—C21	73.67 (17)
N1—C2—C3—C16	117.56 (13)	C2—C3—C16—C21	−164.37 (13)
O1—C2—C3—C10	60.7 (2)	C21—C16—C17—C18	1.6 (2)
N1—C2—C3—C10	−118.01 (13)	C3—C16—C17—C18	−177.83 (14)
C1—S1—C4—C5	−166.01 (13)	C16—C17—C18—C19	−0.6 (2)
C2—N1—C6—C7	−102.35 (18)	C17—C18—C19—C20	−0.8 (3)
C1—N1—C6—C7	76.9 (2)	C18—C19—C20—C21	1.0 (3)
N1—C6—C7—C8	58.9 (2)	C19—C20—C21—C16	0.0 (3)
N1—C6—C7—C9	−176.20 (16)	C17—C16—C21—C20	−1.3 (2)
N2—C3—C10—C11	−22.7 (2)	C3—C16—C21—C20	178.12 (15)

### 2-(Ethylsulfanyl)-3-(2-methylpropyl)-5,5-diphenyl-4,5-dihydro-3H-imidazol-4-one Hydrogen-bond geometry (Å, º)

*Cg*2 and *Cg*3 are the centroids of the C10–C15 and C16–C21 benzene rings, respectively.

**Table:**

*D*—H···*A*	*D*—H	H···*A*	*D*···*A*	*D*—H···*A*
C5—H5*A*···C*g*2^i^	0.98	2.90	3.670 (2)	136
C5—H5*C*···O1^ii^	0.98	2.57	3.487 (2)	156
C14—H14···C*g*3^iii^	0.95	2.79	3.6536 (17)	151

Symmetry codes: (i) *x*−3/2, −*y*+1/2, *z*−3/2; (ii) *x*−1/2, −*y*+3/2, *z*−1/2; (iii) −*x*+1, −*y*+1, −*z*+2.
